# Syndecan-1 as an immunogene in Triple-negative breast cancer: regulation tumor-infiltrating lymphocyte in the tumor microenviroment and EMT by TGFb1/Smad pathway

**DOI:** 10.1186/s12935-023-02917-7

**Published:** 2023-04-17

**Authors:** Ying Zhong, Fangyuan Li, Sumei Zhang, Zhenli Yang, Xinyu Ren, Xi Cao, Yali Xu, Dan Guo, Yidong Zhou, Feng Mao, Songjie Shen, Qiang Sun

**Affiliations:** 1 Department of Breast Disease, Peking Union Medical College Hospital, No. 1 Shuaifuyuan, Wangfujing, Beijing, 100730 China; 2 Medical Research Central, Peking Union Medical College Hospital, Chinese Academy of Medical Sciences, No. 1 Shuaifuyuan, Wangfujing, Beijing, 100730 China; 3 Clinical Biobank, Peking Union Medical College Hospital, Chinese Academy of Medical Sciences, No. 1 Shuaifuyuan, Wangfujing, Beijing, 100730 China; 4 Cell Resource Center, Institute of Basic Medical Sciences, Chinese Academy of Medical Sciences (CAMS) & School of Basic Medicine, Peking Union Medical College, No. 5 Dongdansantiao, Dongcheng, Beijing, 100730 China; 5 Department of Pathology, Peking Union Medical College Hospital, No. 1 Shuaifuyuan, Wangfujing, Beijing, 100730 China

**Keywords:** SDC1, Triple negative breast cancer, Prognosis, TGFb1-Smad, Tumor-infiltrating lymphocyte

## Abstract

**Background:**

Immune checkpoint inhibitors are the most studied forms of immunotherapy for triple-negative breast cancer (TNBC). The Cancer Genome Map (TCGA) and METABRIC project provide large-scale cancer samples that can be used for comprehensive and reliable immunity-related gene research.

**Methods:**

We analyzed data from TCGA and METABRIC and established an immunity-related gene prognosis model for breast cancer. The SDC1 expression in tumor and cancer associated fibroblasts (CAFs) was then observed in 282 TNBC patients by immunohistochemistry. The effects of SDC1 on MDA-MB-231 proliferation, migration and invasion were evaluated. Qualitative real-time PCR and western blotting were performed to identify mRNA and protein expression, respectively.

**Results:**

*SDC1*, as a key immunity-related gene, was significantly correlated with survival in the TCGA and METABRIC databases, while *SDC1* was found to be highly expressed in TNBC in the METABRIC database. In the TNBC cohort, patients with high SDC1 expression in tumor cells and low expression in CAFs had significantly lower disease-free survival (DFS) and fewer tumor-infiltrating lymphocytes (TILs). The downregulation of SDC1 decreased the proliferation of MDA-MB-231, while promoting the migration of MDA-MB-231 cells by reducing the gene expression of E-cadherin and TGFb1 and activating p-Smad2 and p-Smad3 expression.

**Conclusion:**

*SDC1* is a key immunity-related gene that is highly expressed TNBC patients. Patients with high SDC1 expression in tumors and low expression in CAFs had poor prognoses and low TILs. Our findings also suggest that SDC1 regulates the migration of MDA-MB-231 breast cancer cells through a TGFb1-Smad and E-cadherin-dependent mechanism.

**Supplementary Information:**

The online version contains supplementary material available at 10.1186/s12935-023-02917-7.

## Introduction

Breast cancer is the most common cancer and the leading cause of cancer-related deaths in women [1]. Approximately 3–10% of new breast cancer cases have distant metastasis at the time of diagnosis. In the early stage, 30–40% of patients develop advanced breast cancer, and the five-year survival rate is only 20% [2].

Based on genotype analyses, breast cancer can be divided into the following molecular types: luminal A, luminal B, HER2, basal-like, and normal-like [3]. Triple-negative breast cancer (TNBC) accounts for 13–20% of all breast cancers and is an invasive subtype [4, 5]. The prognosis of patients with TNBC is poor, partly due to the lack of effective targeted drugs; thus, the mainstay of treatment has been traditional chemotherapy [5–8]. Gene expression profiles have shown that TNBC subtypes were widely distributed from basal to luminal, and most, but not all, TNBC subtypes expressed basal-like properties [4, 9]. Many gene changes have been observed in TNBC, including *TP53*, *PIK3CA*, *BRCA1*, and *BRCA2* mutations [10]. *BRCA1*/*2* mutations have been found in 11% of TNBC patients [11]. In addition, TNBC has one of the highest mutation rates of all breast cancer subtypes [12]. TNBC has abundant gene ontologies in immune cell processes including immune cell signaling pathways [13]. It is also considered to be the most immunogenic subtype with higher tumor infiltrating lymphocyte (TIL) density than other subtypes, which is associated with improved prognosis [14–17] and TIL density can predict the effect of neoadjuvant therapy [18, 19]. Stromal TILs were significantly associated with the OS in patients with cancer [20–23]. Different immune cell types have been investigated in the prognosis and immunotherapy of a variety of tumors [21, 24–27]. Higher TILs are associated with improved prognosis of HER2-positive breast cancer and TNBC patients. For every 10% increase in TILs, recurrence and mortality decreased by 15–25% [14–17]. These data suggest that TNBC is a highly heterogeneous and immunogenic tumor type; therefore, finding new immunity-related targets is critical for prognosis and effective treatment.

We systematically analyzed the expression of immunity-related genes and clinical outcomes of breast cancer patients in the TCGA database, aiming to screen for immunity-related genes related to prognosis and develop a personalized immune prognosis gene panel for breast cancer patients. We further explored the key immune genes, observed their relationship with the prognosis of TNBC and searched possible mechanisms to find new immunotherapeutic targets for TNBC.

## Materials and methods

### Clinical samples and data collection

Transcriptome RNA sequencing data of 111 normal and 1053 breast cancer samples were downloaded from the TCGA data portal (https://portal.gdc.cancer.gov/repository), and the list of immunity-related genes were obtained through ImmPort [28] (https://www.immport.org/).

### Differential gene analysis

R (3.6.3) [29] was used to compare all differentially expressed genes (DEGs) between breast cancer and normal tissues, and the false discovery rate of < 0.05 and the log_2_ |fold change|> 1 were set as the critical values. Then, the immunity-related DEGs for breast cancer and normal tissues were extracted and compared using R 3.6.3 and the limma, pheatmap, BiocManager, surviva, and survivalROC packages. To explore the potential molecular mechanism of immunity-related DEGs, functional enrichment analysis was carried out using Gene Ontology (GO) pathway analysis (http://www.gsea-msigdb.org/gsea/login.jsp).

### Construction of an immunity-related gene prognosis model

The clinical information including the age, tumor-node-metastasis (TNM) stage, tumor size, lymph node metastasis, distant metastasis, and long-term follow-up results of the patients were also extracted from the TCGA database. The association between survival outcome and immunity-related gene expression in breast cancer patients was analyzed to find out the immunity-related genes that were significantly associated with prognosis. By combining the key immunity-related genes obtained with the outcome information of breast cancer patients from the TCGA clinical database, we constructed an immunity-related gene prognosis model by multivariate analysis. The Cox regression coefficient was used to analyze the relationship between gene expression and survival outcome.

### Analysis of clinical characteristics and immune cells associated with immunity-related gene prognosis model

The patients in the TCGA clinical database were divided into high- and low-risk groups, according to the median risk value calculated by the model to evaluate its prognostic value. Gene expression data were analyzed again using the TIMER [30] database (https://cistrome.shinyapps.io/timer/), which included 10,897 samples of 32 cancer types of TCGA. The breast cancer sample data downloaded from the TCGA database intersected with the samples from the TIMER database, and the contents of six immune cell types in the intersections were obtained, including B, CD4T^+^, CD8T^+^, macrophage, neutrophil, and dendritic cells. We observed the relationship between immune cell infiltration and the risk value in each sample calculated by the immunity-related gene prognosis model.

### Analysis of clinical and molecular characteristics associated with key immunity-related genes

We verified the key immunity-related genes in the model using 1093 breast cancer patients with clinical data and survival outcomes in the METABRIC database. The associations were analyzed between key immunity-related genes and survival in the TCGA and METABRIC databases. The key immunity-related genes and clinical features were also observed. *SDC1* was selected for subsequent research because it was significantly correlated with survival, TNBC, age, and lymph node metastasis.

### Clinical samples and immunohistochemistry in TNBC cohort

The tissue microarray (TMA) was made using formalin-fixed paraffin-embedded tumor samples from 282 consecutive triple-negative breast cancer patients in Peking Union Medical College Hospital (PUMCH). Each tumor specimen contained the tumor and tumor stroma. All the patients had been operated in PUMCH from 2011 to 2014 and had tumors in stages I–III. All the pathological characteristics including tumor size, lymph nodes, tumor grade, hormone receptor (HR) status, and HER2 status were observed. The median follow-up time was 69 months (1–104 months). The TILs in each sample were counted according to the publication *A Practical Review for Pathologists and Proposal* [31]. All TMA were stained for SDC1 (prediluted, Jinqiaoyatu, Beijing, China), CD3 (PA0553, prediluted; Leica Microsystems, Shanghai, China), CD4 (PA0427, prediluted; Leica Microsystems), CD8 (PA0183, prediluted; Leica Microsystems), CD19 (ZA-0569, Prediluted; Zhongshan Golden Bridge Biotechnology, Beijing, China).

The expression of SDC1 in tumor cells, paracancerous normal mammary duct cells (PNMDCs) and cancer-associated fibroblast (CAFs), as well as frequencies of CD3, CD4, CD8, CD19, and TILs were independently measured by two pathologists. The expression of SDC1 in tumor cells and PNMDCs were observed according to histochemical scoring (H-score) [32]. H-scores of 0–49, 50–99, 100–199, and 200–300 were classified as 0, 1 + , 2 + , and 3 + , respectively. The patients with classifications of 0, 1 + , 2 + were defined as belonging to the negative group, while patients with a classification of 3 + were defined as being in the positive group for tumor cells and PNMDCs. The patients were divided into negative group and positive group for CAFs according to the presence or absence of staining of SDC1. The patients were divided into high and low groups according to a TIL median of 5%, CD3^+^ TIL median of 20%, CD4^+^ TIL median of 10%, CD8^+^ TIL median of 10%, and CD19^+^ TIL median of 1%.

### Cell culture

Human breast cancer cell line MDA-MB-231 was obtained from the Chinese Academy of Medical Sciences (ATCC^®^HTB-26^™^, Beijing, China). The cells were cultured in L15 medium containing 20% fetal bovine serum at 37 °C and in normal air. Lentiviral particles with SDC1 overexpression or shRNA expression and their negative controls were obtained from GenePharma (Shanghai, China). MDA-MB-231 cells were incubated in a 24-well plate for 24 h and then transduced with the shRNA-expressing lentivirus, SDC1 overexpression, or their control empty lentivirus for 48 h. Puromycin (LABLEAD Biotech, Beijing, China) was used to select cells for two weeks. Then the stably transfected *SDC1* knockdown (5′-GGAGCAGGACTTCACCTTTGA-3ʹ) cell lines (LV3-NC and LV3-SDC1), overexpression (6382, NM_001006946.2, Additional file 1: Table S1) cell lines (LV5-NC and LV5-SDC1) based on MBA-MD-231 and their control empty cells were cultured in RPMI-1640 (AQ11875, Beijing Aoqing Biotechnology, China) supplemented with 10% fetal bovine serum (Gibco; Thermo Fisher Scientific, Inc., Waltham, MA, USA) at 37 °C in an incubator with 5% CO_2_.

### Cell Counting Kit-8 (CCK-8)

The cells were seeded in the 96-well plates (5000 cells/well) and cultured for 24 h, 48 h, 72 h. Then, 10 μL of CCK-8 reagent (CK04-500 T, DOJINDOi) was added to the 100 μL culture medium. After 4 h incubation in a 37 °C incubator, the absorbance at 450 nm was measured using an EPOCH2 Microwell Plate Spectrophotometer (BioTek, Winooski, VT).

### Transwell assay

The cells (4 × 10^4^ cells/well) were plated in a transwell chamber with 8 μM pore size in 200 μL serum-free RPMI-1640, and the lower chamber was filled with 600 μL DMEM with 10% PBS. After 24 h incubation, the cells on the upper side of the chamber were removed and the migrated cells were fixed with 4% paraformaldehyde and stained with crystal violet. Number of migrated cells were measured using Axio Imager A2 microscope (Carl Zeiss Microscopy GmbH).

### Quantitative real-time PCR (RT-qPCR)

TRIzol^™^ Reagent (15596026, ThermoFisher Scientific) was used for total RNA isolation. Reverse transcription into cDNA was done using PrimeScript^™^ RT Master Mix (Perfect Real Time) Kit (RR036A, TAKARA, Kusatsu, Japan). RT-qPCR was performed by Fast SYBR^®^ Green Master Mix (4385612, ThermoFisher Scientific) on QuantStudio^™^ 7 Flex Real-Time PCR System (4485701, ThermoFisher Scientific). The relative level was calculated using the 2^−△△Ct^ method, and expressed as the ratio of GAPDH. The primer sequences are listed in Additional file 2: Table S2.

### Western blot

The protein extraction was carried out by RIPA lysis buffer (AQ521, Beijing AOqing Biotechology, China) containing ProtLytic Protease Inhibitor Cocktail (P001, New Cell & Molecular Biotech, Shanghai, China). The cell lysates were then resolved by NuPAGE™ (4–12%) Bis–Tris PAGE (NP0322BOX, ThermoFisher Scientific) and transferred onto PVDF membranes. After blocking, the membranes were incubated overnight with primary antibodies against SDC1 (ab128936, 1:1000; Abcam, Cambridge, UK), Smad2 (5339, 1:1000; CST, Danvers, MA, USA), Phospho-Smad2 (18338, 1:1000; CST), anti-smad3 (ab40854, 1:1000; Abcam), Phospho-Smad3 (9520, 1:1000; Abcam), E-Cadherin (3195, 1:1000; CST), N-Cadherin (13116, 1:1000; CST), Vimentin (5741, 1:1000; CST), TGF beta 1 (ab215715, 1:1000; Abcam) and HRP-conjugated GAPDH Monoclonal antibody (HRP-60004, 1:10000; ProteinTech) at 4 °C. Then the membranes were washed three times and incubated with HRP-conjugated Affinipure Goat Anti-Rabbit IgG (H + L) (SA00001-2, 1:10000; ProteinTech, Chicago, IL, USA) for 1 h at 24 °C. The NcmECL Ultra Kit (P10300, New Cell and Molecular Biotech) was added, the proteins were visualized by Amersham Imager 680 (Beijing LABAID Science and Technology. LTD, China) and were evaluated using ImageJ 1.8.0.

### Statistics

R3.6.3 Statistical Software and SPSS 23.0 were used to perform statistical analyses. The difference in gene expression among clinical samples was analyzed by an independent *t*-test. The R “survival” package and Kaplan–Meier method in SPSS were used for survival analysis. We used the survival package to perform a multivariate Cox analysis to establish an immunity-related gene prognosis model. The area under the curve was calculated after creating the ROC curve [33] using the survival ROC package to test the performance of the prognotic model. R was used to draw the charts for the bioinformatic analysis. A *P* < 0.05 was considered statistically significant.

## Results

### Obtaining differential genes and construction of an immunity-related gene prognosis model

**Fig. 1:**
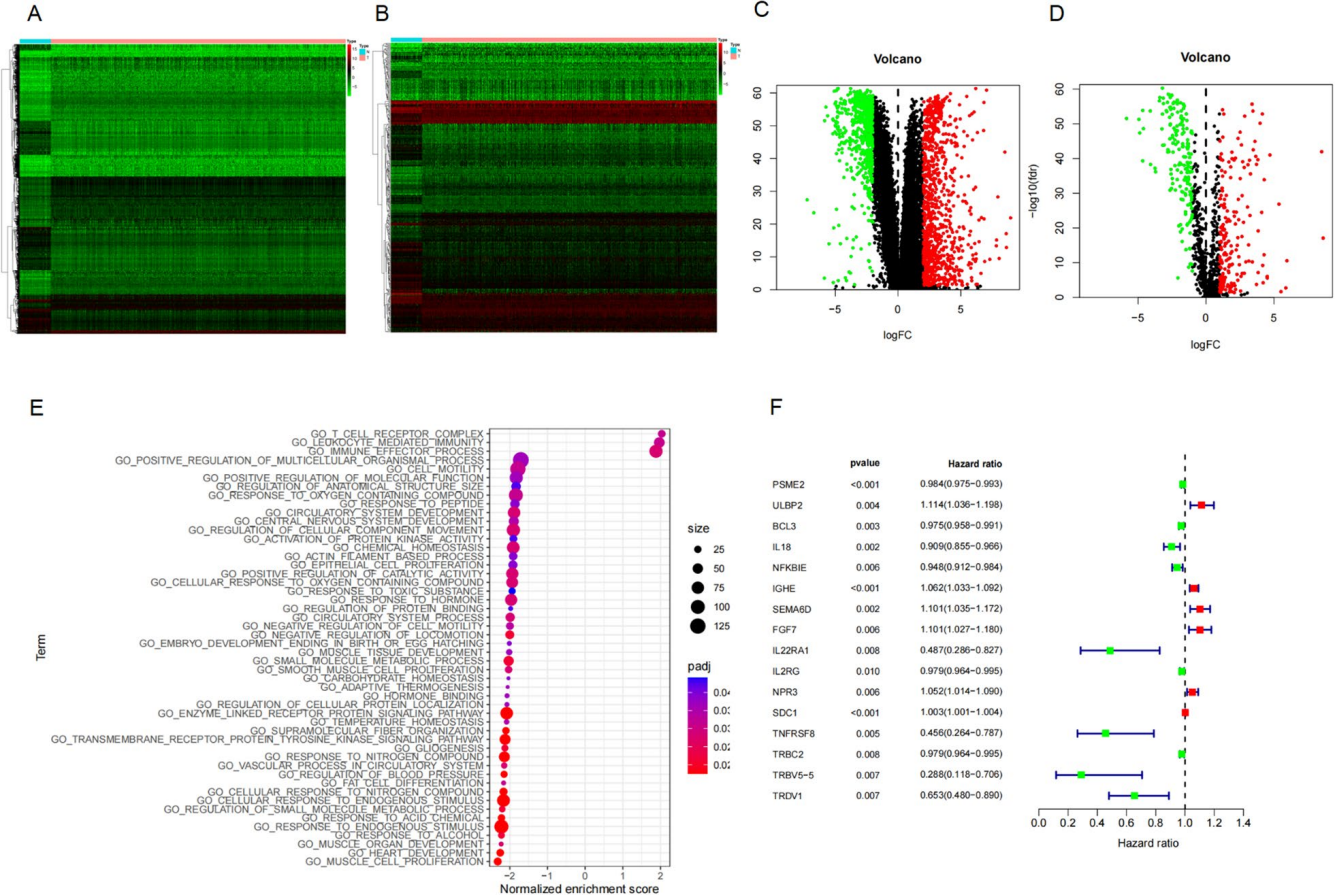
**A** Heatmap and** C** volcano plot showing differentially expressed genes between breast cancer tissue and normal tissue in the TCGA dataset. Red dots represent differentially up-regulated genes, green dots represent differentially downregulated genes and black dots represent no differentially expressed genes. **B** Heatmap and **D** volcano plot showed different immune-related genes between breast cancer tissue and normal tissue in the TCGA dataset. Red dots represent differentially up-regulated genes, green dots represent differentially downregulated genes and black dots represent no differentially expressed genes. **E** The most significant genomes pathways by gene functional enrichment in immune-related genes. **F** Sixteen immunity-related differentially expressed genes significantly associated with overall survival in the TCGA datasets

We extracted 56,753 genes from the TCGA database, including 942 upregulated and 590 downregulated genes (Fig. 1A, C). A total of 377 immunity-related DEGs were extracted after intersection with the ImmPort database, including 203 upregulated and 174 downregulated genes (Fig. 1B, D). Enrichment analysis showed that the immune inflammatory pathway was one of the most significantly enriched pathways (Table 1). T cell receptor complex, leukocyte-mediated immunity, and immune effector process pathways had high enrichment scores (Fig. 1E). Among them, we found that 16 immunity-related DEGs were significantly associated with overall survival (OS) in breast cancer patients (Fig. 1F) and their average differences are shown in Table 2. To better predict the prognosis of patients with breast cancer, an immunity-related gene prognosis model was constructed by multivariate Cox regression analysis. The formula was as follows: [Expression level of *ULBP2* * 0.1160] + [Expression level of *BCL3* *(− 0.0166)] + [Expression level of *IL18* * (− 0.0696)] + [Expression level of IGHE* 0.0602] + [Expression level of SEMA6D* 0.1086] + [Expression level of *FGF7* * 0.0919] + [Expression Level of *IL22RA1* * (-0.7509)] + [Expression level of *NPR3* * 0.0445] + [Expression level of *SDC1* * 0.0014] + [Expression level of *TRDV1* * (-0.2995)].

**Table 1:** The most significant pathway by functional Enrichment analysis

Pathway	padj	ES	NES	Size
GO_RESPONSE_TO_ACID_CHEMICAL	0.01765	− 0.50902	− 2.22169	25
GO_SUPRAMOLECULAR_FIBER_ORGANIZATION	0.01765	− 0.48201	− 2.10382	25
GO_REGULATION_OF_BLOOD_PRESSURE	0.01765	− 0.50048	− 2.15203	24
GO_HEART_DEVELOPMENT	0.01765	− 0.50506	− 2.25404	27
GO_MUSCLE_CELL_PROLIFERATION	0.01765	− 0.50868	− 2.32071	29
GO_CELLULAR_RESPONSE_TO_NITROGEN_COMPOUND	0.01765	− 0.47024	− 2.16689	30
GO_RESPONSE_TO_NITROGEN_COMPOUND	0.01765	− 0.39294	− 2.14571	56
GO_TRANSMEMBRANE_RECEPTOR_PROTEIN_TYROSINE_KINASE_SIGNALING_PATHWAY	0.01765	− 0.38748	− 2.12596	57
GO_ENZYME_LINKED_RECEPTOR_PROTEIN_SIGNALING_PATHWAY	0.01765	− 0.35315	− 2.08362	79
GO_CELLULAR_RESPONSE_TO_ENDOGENOUS_STIMULUS	0.01765	− 0.36486	− 2.16811	81
GO_RESPONSE_TO_ENDOGENOUS_STIMULUS	0.01765	− 0.35867	− 2.2226	101
GO_REGULATION_OF_SMALL_MOLECULE_METABOLIC_PROCESS	0.021166	− 0.53436	− 2.20154	21
GO_RESPONSE_TO_ALCOHOL	0.021166	− 0.53187	− 2.22304	22
GO_GLIOGENESIS	0.021166	− 0.50307	− 2.12947	23
GO_NEGATIVE_REGULATION_OF_LOCOMOTION	0.021166	− 0.41126	− 2.0009	36
GO_SMALL_MOLECULE_METABOLIC_PROCESS	0.021166	− 0.38572	− 2.03119	48
GO_RESPONSE_TO_HORMONE	0.027985	− 0.33928	− 1.96476	72
GO_POSITIVE_REGULATION_OF_CATALYTIC_ACTIVITY	0.027985	− 0.3335	− 1.93399	73
GO_CHEMICAL_HOMEOSTASIS	0.027985	− 0.32243	− 1.90815	80
GO_MUSCLE_ORGAN_DEVELOPMENT	0.027985	− 0.59343	− 2.22904	16
GO_VASCULAR_PROCESS_IN_CIRCULATORY_SYSTEM	0.027985	− 0.5305	− 2.14832	20
GO_SMOOTH_MUSCLE_CELL_PROLIFERATION	0.027985	− 0.45941	− 2.03232	26
GO_CIRCULATORY_SYSTEM_PROCESS	0.027985	− 0.39925	− 1.98962	40
GO_IMMUNE_EFFECTOR_PROCESS	0.027985	0.348071	1.880554	87
GO_CELLULAR_RESPONSE_TO_OXYGEN_CONTAINING_COMPOUND	0.027985	− 0.3392	− 1.93643	67
GO_CIRCULATORY_SYSTEM_DEVELOPMENT	0.027985	− 0.32118	− 1.88674	77
GO_REGULATION_OF_CELLULAR_COMPONENT_MOVEMENT	0.027985	− 0.31481	− 1.90474	89
GO_FAT_CELL_DIFFERENTIATION	0.031576	− 0.57632	− 2.16475	16
GO_LEUKOCYTE_MEDIATED_IMMUNITY	0.031576	0.402027	1.969224	54
GO_RESPONSE_TO_OXYGEN_CONTAINING_COMPOUND	0.031576	− 0.29632	− 1.83935	100

**Table 2:** Immunity-related DEGs significantly associated with overall survival

ID	conMean	treatMean	logFC	pValue
ULBP2	0.313465	1.033828	1.72162	2.25E−10
BCL3	10.86832	21.77691	1.002671	7.40E−30
IL18	1.871154	4.372457	1.224516	1.01E−20
NFKBIE	4.064862	8.77383	1.11	7.16E−31
IGHE	0.073772	1.261696	4.096154	7.32E−26
SEMA6D	3.861534	1.204644	− 1.68057	1.06E−50
FGF7	4.864147	1.519639	− 1.67846	2.22E−45
IL22RA1	0.824342	0.403413	− 1.03098	1.60E−24
IL2RG	5.736202	12.19136	1.087691	0.000147
NPR3	6.301688	1.713778	− 1.87856	4.06E−20
SDC1	33.2673	116.7269	1.81096	4.05E−36
TNFRSF8	0.946059	0.421308	− 1.16705	4.83E−31
TRBC2	5.501714	11.98817	1.123659	0.00186
TRBV5-5	0.09757	0.21412	1.133913	0.001259
TRDV1	0.226715	0.616004	1.442061	0.005183
PSME2	17.71725	41.26326	1.219703	2.58E−41

### Correlation between immunity-related gene model and clinical features

**Fig. 2:**
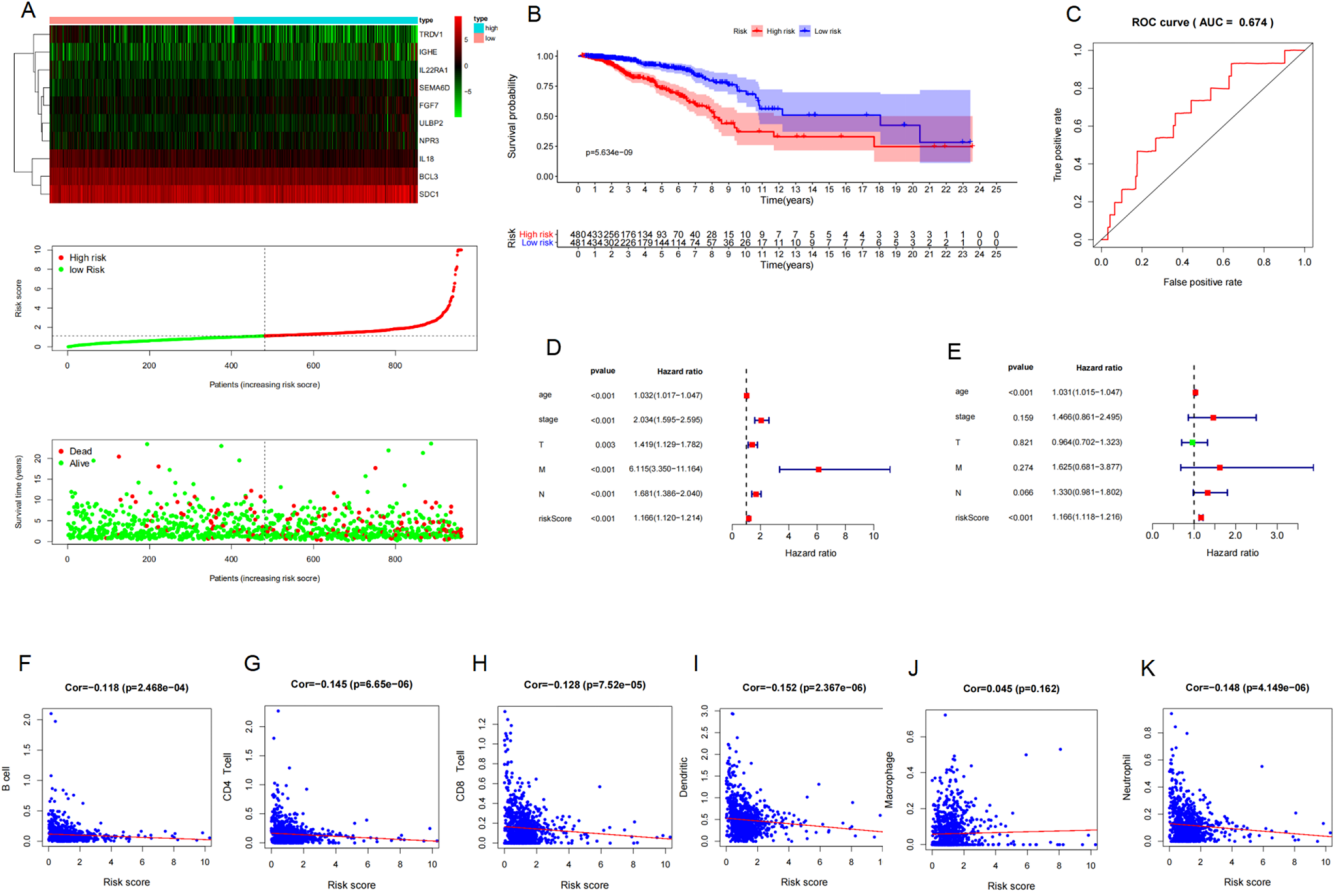
**A** The survival outcome and **B** the overall survival between high risk and low-risk patients distinguished by immunity-related gene model. **C** The prognostic value of the model by receiver operating characteristic (ROC) curve. Univariate analysis (**D**) and multivariate analysis (**E**) of immunity-related gene prognosis model and distant metastasis in the METABRIC datasets. The relationships between B cell infiltration (**F**), CD4^+^ T cell infiltration (**G**), CD8^+^ T cell infiltration (**H**), dendritic infiltration (**I**), macrophage infiltration (**J**), neutrophil infiltration (**K**) and the risk value by the immunity-related gene prognosis model

The model was able to distinguish breast cancer patients into high- and low-risk of death groups, according to the survival outcome of patients (Fig. 2A, B). The area under the curve of the model was 0.674 (Fig. 2C), which indicated that the model could predict the prognosis of breast cancer patients to an extent. Univariate and multivariate Cox analyses showed that the immunity-related gene prognosis model was a good prognostic factor, independent of age, pathological stage, tumor stage, lymph node metastasis status, and distant metastasis status (Fig. 2D, E). The relationship of each sample between immune cell infiltration and the risk value was calculated by the model in the TCGA database (Fig. 2F–K). Patients with high-risk scores had significantly lower immune cell infiltration including B, CD4T^+^, CD8T^+^, macrophage, neutrophil, and dendritic cells.

### *SDC1* association with survival and triple negative breast cancer patients

**Fig. 3:**
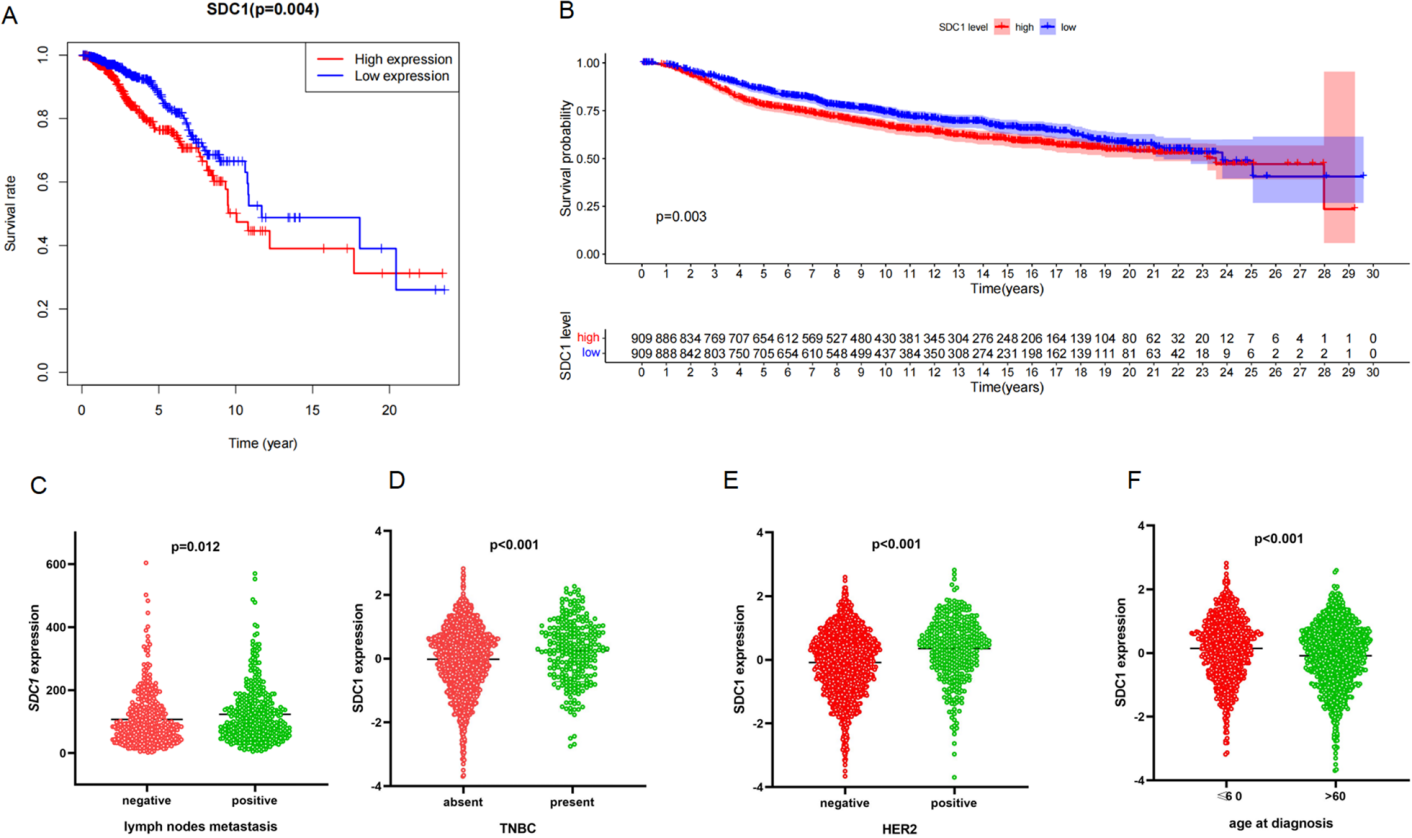
**A** The overall survival and **B** disease-free survival in METABRIC datasets between patients with high expression and low expression of *SDC1*. **C** *SDC1* expression in patients with or without lymph node metastasis in the TCGA dataset. In the METABRIC dataset, **D** *SDC1* expression in patients with TNBC and in other patients, **E** *SDC1* expression in HER2-positive patients and HER2-negative patients, **F** *SDC1* expression in patients aged ≤ 60 years old and > 60 years old

We employed our model to analyze the correlation between immune-related genes and survival of patients with breast cancer, using data on the clinical features and survival outcomes of 1053 and 1093 patients in the TCGA and METABRIC database, respectively. We found *SDC1* was significantly associated with overall survival (Fig. 3A), and lymph node metastasis (Fig. 3C) in TCGA database. We also found that *SDC1* was also significantly associated with disease-free survival (DFS) (Fig. 3B). In addition, *SDC1* was significantly higher in TNBC patients and HER2-positive patients (Fig. 3D, E). *SDC1* was also found to be higher in patients ≤ 60 years old than in patients > 60 years old (Fig. 3F).

### SDC1 association with survival and recurrence in TNBC patients

**Fig. 4:**
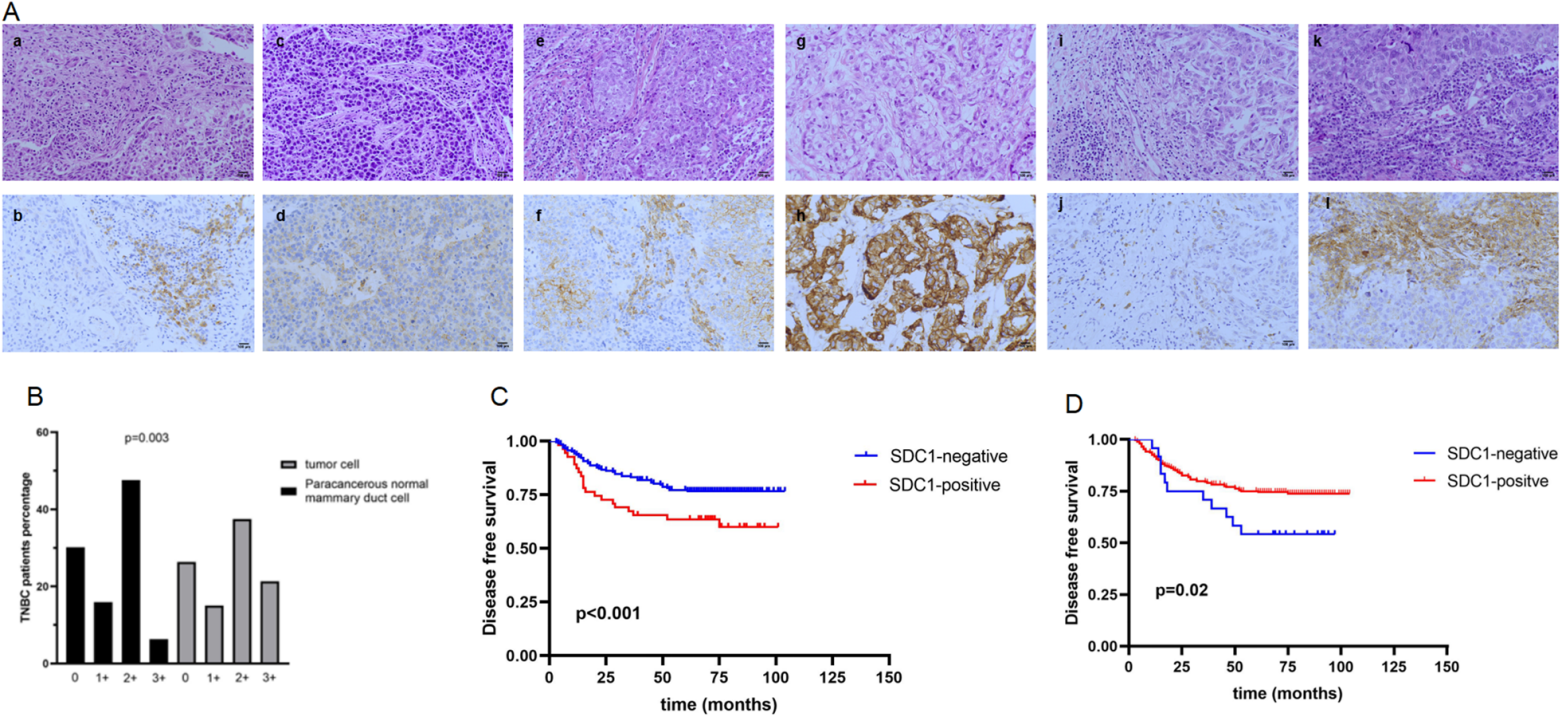
**A** Hematoxylin and eosin (HE) staining and immunohistochemistry (IHC) of SDC1 expression in TNBC patients. SDC1-negative expression on tumor cell by (**a**) HE staining and **b** IHC (original magnification, × 200). SDC1 1 + expression on tumor cells by (**c**) HE staining and **d** IHC (original magnification, × 200). SDC1 2 + expression on tumor cells by (**e**) HE staining and **f** IHC (original magnification, × 200). SDC1 3 + expression on tumor cells by (**g**) HE staining and **h** IHC (original magnification, × 200). SDC1-negative expression in cancer-associated fibroblasts (CAFs) by (**i**) HE staining and **j** IHC (original magnification, × 200). SDC1-positive expression on CAF by (**k**) HE staining and **l** IHC (original magnification, × 200). **B** The percentage of TNBC patients with the expression of SDC1 in tumor cells and in paracancerous normal mammary duct cells (PNMDCs). Disease-free survival between SDC1-positive patients and SDC1-negative patients in tumor cells **C** and in CAFs **D** in the TNBC cohort

We analyzed the 282 TNBC patients treated at PUMCH who had clinical features and survival outcomes. We observed SDC1 expression in tumor cell, PNMDC, and CAF by immunohistochemistry (IHC) (Fig. 4A), and the characteristics of these patients are shown in Table 3. The proportion of patients with positive expression of SDC1 in tumor cells was higher than those patients in PNMDC (Fig. 4B, p = 0.003). The patients with positive expression of SDC1 in tumor cells had significantly lower DFS compared to patients with negative SDC1 expression in tumor cells (Fig. 4C, p < 0.001). The patients with negative expression of SDC1 in CAF had significantly lower DFS compared with the patients with positive SDC1 expression in CAFs (Fig. 4D, p = 0.02). In univariate Cox proportional hazards regression analysis, tumor size, positive lymph nodes, positive expression of SDC1 in tumor cells, and negative expression of SDC1 in CAF were significantly associated with distant recurrence and in multivariate Cox proportional hazards regression analysis, positive lymph nodes, negative expression of SDC1 in CAF were significantly associated with distant recurrence (Table 4).

**Table 3:** Characteristics of TNBC patients

Characteristics	No	%
Age median(range)	49 (25–79)	
Menses		
Premenopausal	150	53.2
Postmenopausal	132	46.8
Grade		
1	1	0.4
2	82	29.1
3	198	70.2
uk	1	0.4
Tumor size		
≤ 2 cm	136	48.2
> 2 cm, ≤ 5 cm	131	46.5
> 5 cm	14	5.0
uk	1	0.4
Positive lymph nodes		
0	169	59.9
1–3	61	21.6
4–9	26	9.2
≥ 10	35	12.4
uk	1	0.4
Ki-67		
≤ 14%	27	9.6
> 14%	253	89.7
uk	2	0.7

**Table 4:** Univariate and multivariate analysis of SDC1 and Clinical characteristic with distant recurrence

Clinical characteristic	Univariable analysis			Multivariable analysis		
	HR	95%CI	*P* value	HR	95%CI	*P* value
Menopause	1.12	0.66–1.90	0.67			
Tumor grade	0.72	0.42–1.25	0.25			
T stage	1.95	1.25–3.07	0.004	1.36	0.78–2.37	0.27
N stage	1.86	1.45–2.37	< 0.001	1.70	1.26–2.29	< 0.001
SDC1 in tumor cell	2.08	1.10–3.91	0.02	1.94	0.92–4.07	0.08
SDC1 in CAF	0.33	0.14–0.79	0.012	0.35	0.13–0.91	0.03

### SDC1 is associated with tumor stromal infiltrating lymphocytes in TNBC patients

**Fig. 5:**
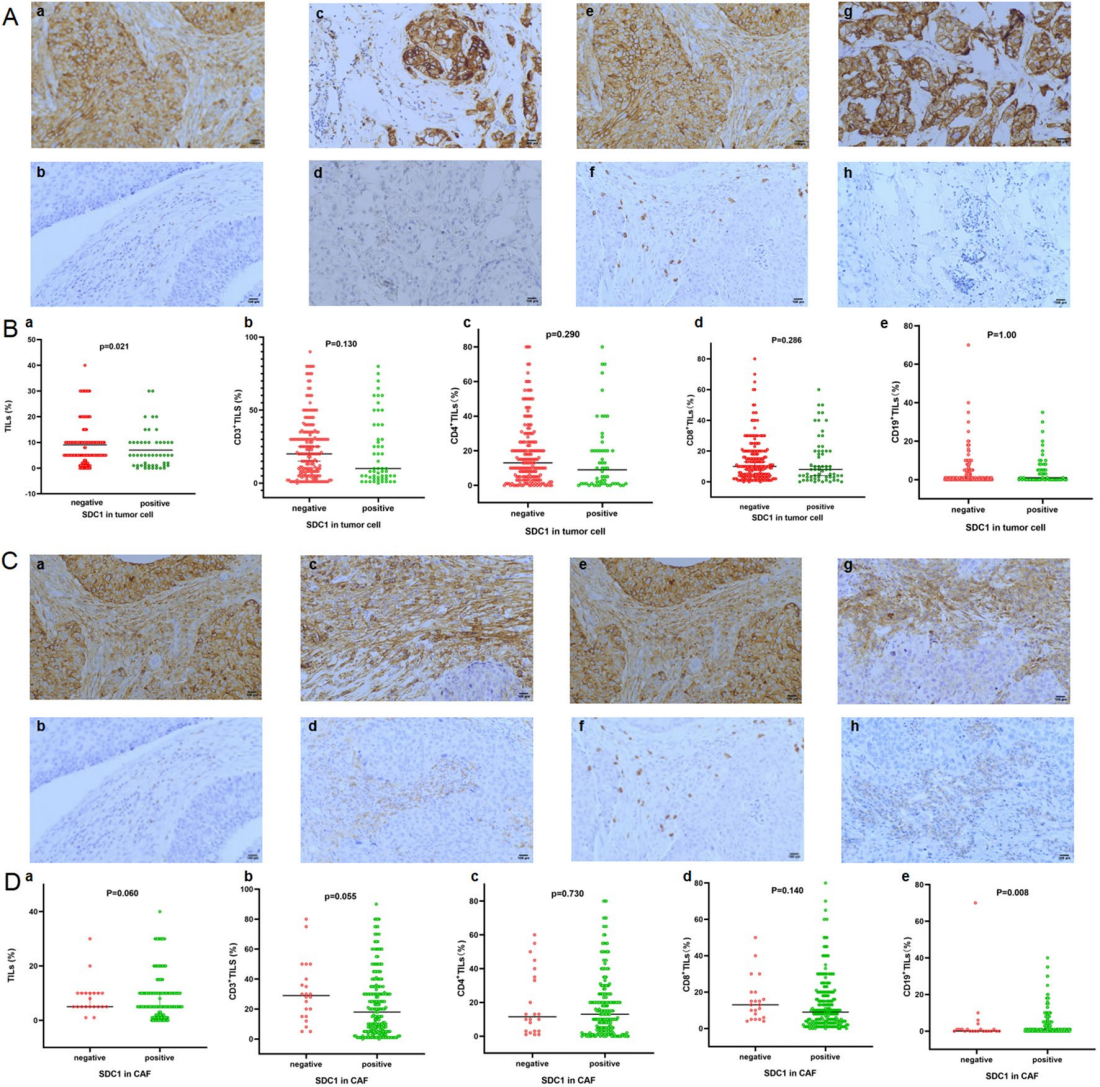
**A** SDC1 expression in tumor cells associated with TILs in the TNBC cohort. **a**, **b** Patients with SDC1-positive expression and a low percentage of CD3^+^ TILs. **c**, **d** Patients with SDC1-positive expression and a low percentage of CD4^+^ TILs. **e**, **f** Patients with SDC1-positive expression and a low percentage of CD8^+^ TILs. **g**, **h** Patients with SDC1-positive expression and a low percentage of CD19^+^ TILs. **B** Frequencies of TILs between patients with SDC1-negative expression and SDC1-positive expression in tumor cells in the TNBC cohort. **a** Frequencies of TILs, **b** CD3^+^ TILs, **c** CD4^+^ TILs, **d** CD8^+^ TILs, **e** CD19^+^ TILs associated with SDC1 expression in tumor cells. **C** SDC1 expression in CAFs associated with TILs in the TNBC cohort. **a**, **b** Patients with SDC1-positive expression and a low percentage of CD3^+^ TILs. **c**, **d** Patients with SDC1-positive expression and a high percentage of CD4^+^ TILs. **e**, **f** Patients with SDC1-positive expression and a low percentage of CD8^+^ TILs. **g**, **h** The patients with SDC1-positive expression and a high percentage of CD19^+^ TILs. **D** Frequencies of TILs between patients with SDC1-negative expression and SDC1-positive expression in CAF in the TNBC cohort. **a** Frequencies of TILs, **b** CD3^+^ TILs, **c** CD4^+^ TILs, **d** CD8^+^ TILs, **e** CD19^+^ TILs associated with SDC1 expression in CAFs

TIL distribution was confirmed by HE, and CD4^+^TILs, CD3^+^TILs, CD8^+^TILs and CD19 ^+^TILs were also observed by IHC in 282 TNBC samples (Fig. 5A, C). The patients with positive expression of SDC1 in tumor cells were associated with lower TIL distribution (Fig. 5B, p = 0.021). CD3 T, CD4 T, CD8 T, and CD19 T cell distributions were lower in patients with positive SDC1 expression than in patients with negative SDC1 expression in tumor cells, albeit there was no significant difference (Fig. 5B). TIL distribution was lower in patients with negative SDC1 expression than patients with positive expression of SDC1 in CAF, although there was no significant difference (Fig. 5D, p = 0.060). CD3 T cells, CD8 T cells were lower in patients with positive expression of SDC1 than those with negative SDC1 expression in CAF (Fig. 5D, p = 0.055, p = 0.140). CD4 T and CD19 T cell counts were higher in patients with positive expression of SDC1 than those with negative expression in CAFs (Fig. 5D, p = 0.730, p = 0.008).

### SDC1 knockdown inhibited the viability in MDA-MB-231 cells

**Fig. 6:**
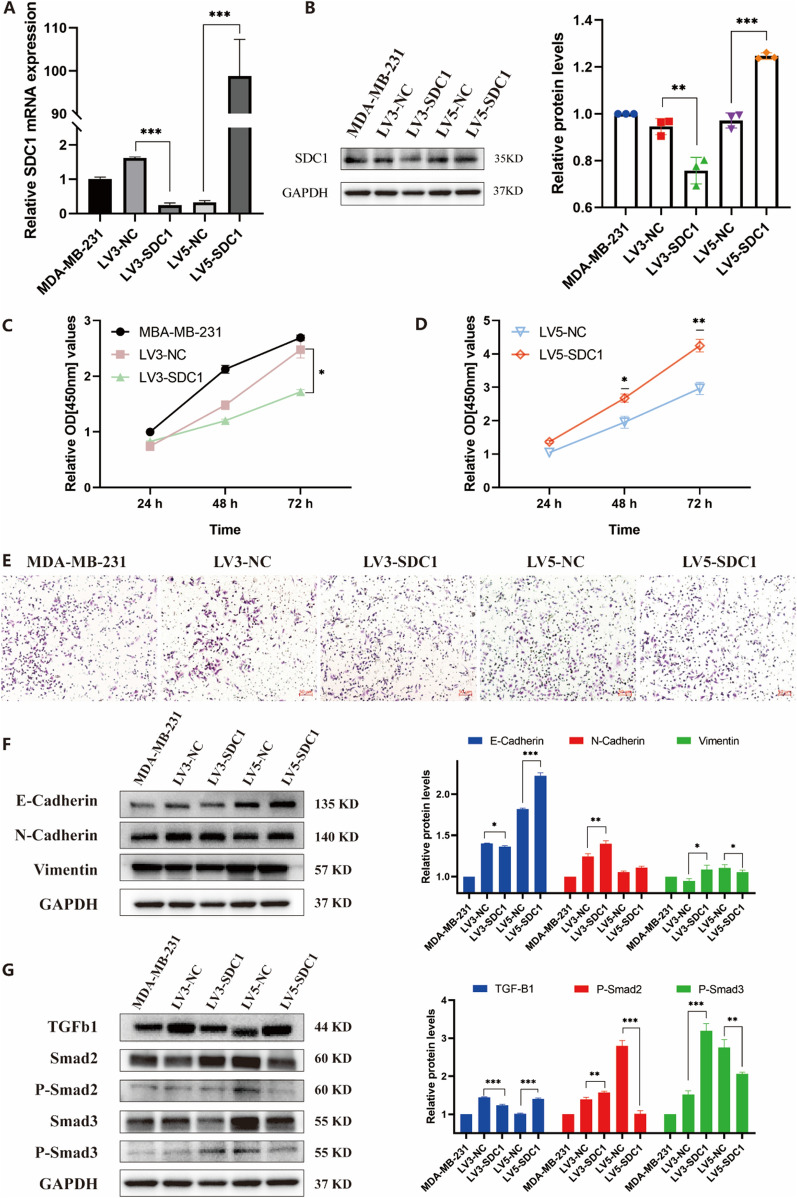
The effect of SDC1 on breast cancer cell proliferation and migration. **A** Relative SDC1 mRNA expression after knockdown and overexpression. **B** Relative SDC1 protein expression after knockdown and overexpression. A CCK-8 assay evaluated the effects of SDC1 on cell proliferation after the **C** knockdown and **D** overexpression of SDC1. **E** Transwell assay determined that SDC1 knockdown promoted the invasion of MDA-MB-231 cells. **F** The protein levels of EMT-related proteins. **G** Protein expression in the TGF-β1/Smad signaling pathway. Results with P < 0.05 were considered statistically significant. An unpaired *t*-test was used for data analysis *p ≤ 0.05, **p ≤ 0.01, ***p ≤ 0.001

To further explore the mechanism of SDC1 in breast cancer, especially in TNBC, we conducted cell experiments to evaluate the effects of SDC1 on breast cancer cell proliferation and migration. First, we constructed the stable knockdown and overexpression cell lines and verified their expression effects. A significant decrease to 15.4% in gene expression of *SDC1* in MDA-MB-231 cell line was observed after *SDC1* knockdown (LV3-SDC1) compared with the NC control (LV3-NC, Fig. 6A, B), while *SDC1* was increased by 302.6 times after SDC1 overexpression (LV5-SDC1) compared to the NC control (LV5-NC, Fig. 6A, B).

The CCK-8 assay was utilized to evaluate the effects of SDC1 on cell proliferation. The cell proliferation decreased significantly after the knockdown of *SDC1* (Fig. 6C), meanwhile the cell viability increased after *SDC1* overexpression (Fig. 6D).

### SDC1 knockdown promoted the invasion of MDA-MB-231 cells

To understand the potential roles of SDC1 in mediating MDA-MB-231 cell motility, a transwell assay was performed (Fig. 6E). The number of cells passing through the aperture was significantly higher in the siSDC1 group (LV3-SDC1) compared with the MDA-MB-231 and LV3-NC groups after 24 h incubation. The 24 h migration rate was decreased in the SDC1 overexpression group (LV5-SDC1) compared with the LV5-NC group.

### *SDC1* affected epithelial–mesenchymal transition (EMT) via regulating the TGF-β1/Smad pathway signaling pathway

Protein expression levels of the epithelial marker E-cadherin was reduced, while those of the interstitial markers N-cadherin and vimentin were significantly increased by *SDC1* knockdown (Fig. 6F). In the meantime, the overexpression of SDC1 increased the protein level of E-cadherin and reduced the protein expression of N-cadherin and vimentin. The results showed that *SDC1* knockdown or overexpression affected the expression of epithelial and interstitial markers, modulating EMT in MDA-MB-231 cells.

Given the correlation between the EMT process and the TGF-β1 pathway, western blotting was used to detect the effect of *SDC1* knockdown or overexpression on the TGF-β1/Smad pathway. After the knockdown of *SDC1*, the expression of TGF-β1 was significantly decreased, while phospho-Smad3 and phospho-Smad2 expression increased. In contrast, overexpression of *SDC1* caused the opposite results (Fig. 6G).

## Discussion

Although early-stage breast cancer can be successfully treated through surgery, chemotherapy, or other treatment, more than 30% of patients will eventually progress to an advanced stage [34]. Advanced breast cancer has poor prognosis, and the long-term survival rate is less than 5% [35]. The 5-year OS for patients with TNBC is 72%, with a life expectancy of only 3.55 years [36]. Immune checkpoint inhibitors have been successfully used to treat cancer [37], and immunotherapy has become the preferred treatment for TNBC patients with PD-L1 positive tumors [38]. Therefore, it is crucial to explore new therapeutic targets and tumor markers related to TNBC immunotherapy.

Although the importance of immunity-related genes in cancer therapy has been confirmed [39–42], there has been no comprehensive analysis of the clinical significance and molecular mechanisms of immunity-related genes in breast cancer patients. Here, we found that SDC1 was highly expressed in TNBC patients at protein and transcriptome levels and could be used as a marker of poor prognosis. We also found that negative expression of SDC1 in CAF was positively related to poor prognosis in TNBC patients. TILs were regulated differently by SDC1 in tumor cells and CAFs. Furthermore, we identified that SDC1 reduction promoted the progression of EMT through TGF-β1/Smad pathway in vitro. Finally, based on the above findings, we propose that an SDC1-associated related immune signature as a predictor and a potential target in TNBC.

The expression and immunoregulatory function of SDC1 in cancers are tumor-specific and their details remain controversial. SDC1 ectodomains attenuate allergic lung inflammation via suppression of CC chemokine-mediated Th2 cell recruitment to the lung [43]. SDC1 suppression in TNBC cell line downregulates IL-6, IL-8 [44, 45]. Heparan sulfate chain shedding from SDC-1 facilitates the release of vascular endothelial growth factor C (VEGF-C) into the medium of hepatocarcinoma cells [46]. VEGF suppresses T-lymphocyte infiltration in the tumor microenvironment through inhibition of NF-κB-induced endothelial activation [47]. VEGF induces T-cell apoptosis during extravasation by leading to FASLG on endothelial cells [48]. SDC1 knockdown in SUM-149 cells promotes Th17 cell expansion via upregulation of IL-23 [49]. CD4^+^ T cells differentiate into Th17 cells through TGFB, while IL6 and TH17 maintain proliferation by IL23 [50]. IL6 trans-signaling enhances T-cell transmigration on tumor vessels [51].

In our TNBC cohort, we found that the expression of SDC1 was significantly higher in tumor cells than in PNMDCs. The positive expression of SDC1 in tumor cells was significantly related to a poor prognosis and lower frequencies of TILs. Negative expression of SDC1 in CAF was significantly related to a poor prognosis and lower frequencies of CD19^+^TILs. We speculated that SDC1 in TNBC cells suppressed T-lymphocyte infiltration by upregulating IL6, IL8, and VEGF, while T cell infiltration was inhibited by downregulation of IL-23. Immune escape may be the reason why TNBC patients with positive expression of SDC1 in tumor cells had a poor prognosis.

CAFs participate in tumor occurrence, development, and drug resistance [52]. Activation of CAFs remodel the extracellular matrix and promotes cancer cell invasion and metastasis [53–55]. Highly invasive human breast cancer cells MDA-MB-231 can enhance SDC1 expression in senescent fibroblasts via the paracrine action of TGF-β [56]. Senescent cells were characterized by exhaustion of SDC1 expression [57]. Fusion of dendritic cells and CAFs produce TNF-α and IL-6 [58]. IL-6 is expressed approximately 100-fold higher in CAFs compared to normal fibroblasts [59]. TNF stimulates T-cell extravasation on tumor vessels [60] and IL6 trans-signaling enhances T-cell transmigration on tumor vessels [51]. Activation of CAF induces CXCL16, which in turn recruits T cells [61]. We believe that senescent CAFs with high expression of SDC1 weaken breast cancer cell invasion and metastasis and decrease cytokine secretion; according, patients in our TNBC cohort with negative expression SDC1 in CAFs had poor prognosis and low frequencies of TILs.

SDC1 has been known to play an important role in the invasion, migration and EMT in cancer cells by regulating ERK/Snail signaling [62–64]. As shown in our study, the cell viability and migration of MDA-MB-231 cells were significantly affected after SDC1 knockdown. Western blot assays showed that SDC1 reduction promoted the progression of EMT through the TGF-β1/Smad pathway. These results demonstrated that SDC1 may regulate epithelial-mesenchymal plasticity in TNBC cells and open up the possibility of mechanistic studies to elucidate the mechanisms involved.

In summary, we constructed an immunity-related gene prognosis model that reliably predicted the prognosis of patients with breast cancer. Among the immunity-related genes, *SDC1* was identified as being closely related to the prognosis of breast cancer. The patients with positive expression of SDC1 in tumor cells had poor prognosis and low frequencies of TILs, while patients with negative expression of SDC1 in CAFs had poor prognosis and high frequencies of TILs. In MDA-MB-231, *SDC1* knockout or overexpression affected cell proliferation and migration. SDC1 overexpression in MDA-MB-231 cells upregulated the expression of E-cadherin and TGF-β1, downregulated the expression of p-SMAD2 and p-SMAD3, and affected the EMT process to regulate migration. We believe that TNBC cells and senescent CAFs with positive expression of SDC1 probably inhibit T cells by regulating cytokines, resulting in immune escape. In our research, the associations between SDC1 in tumor cells and CAFs with TILs and related cytokines need to be further confirmed in in vivo and in vitro studies.

## Conclusion

We identified SDC1 as a potential immune-therapeutic target in TNBC through bioinformatics analysis, and the high expression of *SDC1* was significantly associated with a poor prognosis.

Additionally, SDC1 was also closely related with TILs in tumor cells and CAFs and may be involved in the regulation of TILs by various means. Furthermore, we demonstrated that SDC1 regulated EMT through the TGF-β1/Smad pathway. Targeting *SDC1* to regulate tumor cells, CAFs, and TILs should be a promising research direction for TNBC treatment.

## Supplementary Information

**Additional file 1: Table S1**. *SDC1* overexpression.

**Additional file 2: Table S2.** List of primers for RT-qPCR.

## Data Availability

The datasets used and/or analyzed during the current study are available from the corresponding author on reasonable request.
