# Supplementary material for: Syndecan-1 as an immunogene in Triple-negative breast cancer: regulation tumor-infiltrating lymphocyte in the tumor microenviroment and EMT by TGFb1/Smad pathway
Source: Cancer Cell Int. 2023 Apr 17;23:76. doi: 10.1186/s12935-023-02917-7 (PMC10111802; doi:10.1186/s12935-023-02917-7)
Supplement: Supplementary file 1 — Additional file 1: Table S1. SDC1 overexpression. [file 12935_2023_2917_MOESM1_ESM.docx]

Table S1 *SDC1* overexpression

Atgaggcgcgcggcgctctggctctggctgtgcgcgctggcgctgagcctgcagccggccctgccgcaaattgtggctactaatttgccccctgaagatcaagatggctctggggatgactctgacaacttctccggctcaggtgcaggtgctttgcaagatatcaccttgtcacagcagaccccctccacttggaaggacacgcagctcctgacggctattcccacgtctccagaacccaccggcctggaggctacagctgcctccacctccaccctgccggctggagaggggcccaaggagggagaggctgtagtcctgccagaagtggagcctggcctcaccgcccgggagcaggaggccaccccccgacccagggagaccacacagctcccgaccactcatcaggcctcaacgaccacagccaccacggcccaggagcccgccacctcccacccccacagggacatgcagcctggccaccatgagacctcaacccctgcaggacccagccaagctgaccttcacactccccacacagaggatggaggtccttctgccaccgagagggctgctgaggatggagcctccagtcagctcccagcagcagagggctctggggagcaggacttcacctttgaaacctcgggggagaatacggctgtagtggccgtggagcctgaccgccggaaccagtccccagtggatcagggggccacgggggcctcacagggcctcctggacaggaaagaggtgctgggaggggtcattgccggaggcctcgtggggctcatctttgctgtgtgcctggtgggtttcatgctgtaccgcatgaagaagaaggacgaaggcagctactccttggaggagccgaaacaagccaacggcggggcctaccagaagcccaccaaacaggaggaattctatgcctga
