# Supplementary material for: Syndecan-1 as an immunogene in Triple-negative breast cancer: regulation tumor-infiltrating lymphocyte in the tumor microenviroment and EMT by TGFb1/Smad pathway
Source: Cancer Cell Int. 2023 Apr 17;23:76. doi: 10.1186/s12935-023-02917-7 (PMC10111802; doi:10.1186/s12935-023-02917-7)
Supplement: Supplementary file 2 — Additional file 2: Table S2. List of primers for RT-qPCR. [file 12935_2023_2917_MOESM2_ESM.docx]

| Primer ID | SYBR Green primers | |
| --- | --- | --- |
|  |  | Sequence |
| SDC1 | Forward | CGTGGGGCTCATCTTTGCT |
|  | Reverse | TGGCTTGTTTCGGCTCCTC |
| TGF-β1 | Forward | CATGGGAGGTGCTCAGTAAA |
|  | Reverse | CAGGCGGAGAAGGCTTAAT |
| GAPDH | Forward | AGAAGGCTGGGGCTCATTTG |
|  | Reverse | AGGGGCCATCCACAGTCTTC |

Table S2 List of primers for RT-qPCR
